# "Hospitals from the Patients' Point of View" in a Provincial Hospital's Infectious Block

**Published:** 1913-08-30

**Authors:** 


### THE EDITOR'S LETTER-BOX.

### "Hospitals from the Patients' Point of View."

IN A PROVINCIAL HOSPITAL'S INFECTIOUS BLOCK.

*To the Editor of* THE HOSPITAL.

SIR,—Seeing the articles in THE HOSPITAL of patients' experiences I thought mine might be acceptable. I trained in a large provincial hospital of 200 beds. Among other diseases nursed enteric and diphtheria were included, that was because the townspeople did not like them going to the borough hospital, there being no resident; more so, it was a good distance out of town, as is usual with isolation hospitals; and, secondly, it did not bear over and above a very good reputation. Enough money was collected by the inhabitants to build and partly equip a block to be set apart for diphtheria, enteric going to the medical wards, male and female, as the case might be.

The diphtheria block consisted of 22 beds, four small wards upstairs for diphtheria, and a like number downstairs for septic cases. I may mention that before going in for my general training I had received my fever training at a large city hospital of 300 beds. It was in my last year of training that I contracted diphtheria. We got in a particularly bad type of disease; we were very rarely without an intubation or tracheotomy case in the wards. The hospital was staffed with four residents, the resident physician having charge of the diphtheria patients and the junior surgeon the septic cases.

There was a system of bells which were rung in a certain manner to indicate which resident was wanted. The same rule applied at night for the nurses all over the building when they required night sister to pay a visit other than her usual one. The residents were sometimes very tiresome, especially if they had all got together in the senior's room enjoying games, music, friends, etc. The bells might be rung for half an hour. Eventually a nurse having to be dispatched; even then they would take their own time, and saunter leisurely up to the ward just on the point of the nurses changing duty, a thing they seemed very fond of doing, then ordering enough treatment to keep all hands going for a couple of hours or more.

The nursing staff was changed every three months night and day. Those going to the diphtheria ward moved bag and baggage, and had no further communication with the hospital until their three months were over, all food, etc., being fetched by the maid. The ward was staffed with a sister who had never seen a diphtheria patient until she got the appointment, and did not know as much about the treatment as some of the probationers. All she seemed to live for was her off-duty time and the coming of the residents, also the best way she could entertain them, *via* sitting on the fireguards and tables, recounting the latest at the theatre, and what she could find out about the building in general. (She was just as isolated as we were.) She was very young, and it was her first sister's post. The patients to her were nothing—I never remember her taking a temperature. If there was a pretty child, and the resident took a little interest in it, she would manage to be amusing it in some way on his next visit. Beside the sister there were two probationers on day duty and one on night.

I was the senior probationer on the day staff when I contracted diphtheria. . . . Meanwhile I had been sent to the diphtheria ward, and was put in among four small cot children; among the number was a very naughty intubation child.

Now the real agony of my illness begins. Our cots had a rod running up the back of the cot which at the top formed a loop, on which the chart board was hung. Every time the child moved, the board went rat-tat against the rod, and when the children were convalescent, jumping about in their cots and throwing balls to one another, the racket was deafening. I frequently asked the sister if the boards could be removed, but, as I was a member of the staff, I was told they could not, and that I was not to be fanciful. She would immediately start playing with the children, and make more noise than before. I could not stand the noise any longer, and laid with my head in my hands for hours. The junior resident proved one of my best friends; he was young, but very humane, and loved by everybody; he asked me why I did it; very reluctantly I told him, still having it in mind I was only a probationer, and that matron could be told I was giving a lot of trouble, which to the nurse means such a lot. He at once ordered the boards to be removed, and at the same time ordered something for my head. We had a small ward with one bed in it; he said I must go in there, but the sister took a delight in singing and playing with the convalescent children in the adjoining wards, so that the lesser of the two evils turned out the greatest. The day nurse treated me with very little consideration, although she was months my junior, and left me to ask for what treatment and attention I wanted. I did not ask her for much, but waited in agony many a time for the return of the night nurse, who was half German, but a kinder and nobler woman I have never met in the nursing profession.

She always settled me up last thing before she went off duty at 9 A.M., and her first call was to me at the same time at night. At her own suggestion she blanket-bathed me, which was a kindness I shall never forget. She did not want to show off her knowledge in any way, but, knowing that I had done private nursing, she always asked me—"Am I doing so and so the proper way and the way you did it?"—especially did she give me my food as daintily as she could. I was only allowed the same as the hospital patients. But there is just a little finishing touch; and that pillow put just at the right angle under your shoulder—it takes only a minute. The day nurse put the tray on the locker, with the unnecessary remark "Is that all you want?" and departed, leaving the tray usually until the next meal—any way, she was always told to take it away.

The residents were very good in supplying me with magazines and daily papers during my convalescence, and frequently peeped in and chatted a minute or two in the course of their rounds. I have since learned the young junior is married and on the staff as an assistant honorary; also that he has a flourishing private practice, which is no doubt due to his kindness to his more unfortunate brethren. The German girl is theatre sister in a well-known private nursing institute; and, last of all, the sister is matron of a large mental hospital—I should say the best place for her. Fortunately she will not come in contact with the patients, the institution being a large one. Since then I have held various positions in hospital, and at the present time I hold the position of matron and superintendent nurse. I have had sick nurses often under my care and personally cared for them, remembering my own unhappy experience.—I am, Sir, yours faithfully,

K. B.

[It is interesting to contrast this account with the article on "My Experiences in a Metropolitan Asylums Board Hospital," which we publish on page 645.—Ed., THE HOSPITAL.]

